# Supplementary material for: The Role of the Maridi Dam in Causing an Onchocerciasis-Associated Epilepsy Epidemic in Maridi, South Sudan: An Epidemiological, Sociological, and Entomological Study
Source: Pathogens. 2020 Apr 24;9(4):315. doi: 10.3390/pathogens9040315 (PMC7238195; doi:10.3390/pathogens9040315)
Supplement: Supplementary file 1 [file pathogens-09-00315-s001.pdf]

Supplementary information

Table S1. Details of sites at which *S. damnosum* larvae or pupae were surveyed in the Maridi River and its tributaries.

Site No.	Date/2019	River	Site	GPS reading	Elevation	Remarks
1	28 Nov.	Itri	Footbridge	N:4° 53′ 43.6″ E: 29° 26′ 59″	699 m	Swampy poor flow
2	29 Nov.	Maridi	Papyrus site	N: 4° 53′ 45.4″ E: 29° 27′ 30.9″	682 m	Papyrus swamp, poor flow
3	29 Nov.	Maridi	Fish net point	N: 4° 53′ 53.7″ E: 29° 27′ 33.5″	693 m	Sluggish flow
4	29 Nov.	Molisikanga	Footbridge	N: 4° 53′ 54.2° E: 29° 27′ 39.6″	697 m	Papyrus swamp, poor flow
5	29 Nov.	Maridi	Spring well	N: 4° 54′ 8.7″ E: 29° 27′ 38.9″	690 m	Papyrus swamp, poor flow
6	29 Nov.	Maridi	Matara footbridge	N: 4° 54 19.7″ E: 29° 27 41.9″	696 m	Sluggish flow, papyrus choked
7	29 Nov.	Marindo	Main bridge	N: 4° 54′ 44.4″ E: 29° 27′ 61.9″	697 m	Emerges from papyrus swamp
8	29 Nov.	Maridi	Maridi town bridge	N: 4° 54′ 55.3″ E: 29° 27′ 42.6″	690 m	Poor flow emerging from swamp
9	30 Nov.	Maridi dam	Balaba village	N: 4° 53′ 21.3″ E: 29° 27′ 58.8″	704 m	Stagnant water with papyrus
10	30 Nov.	Balaba	Yei road crossing	N:4° 53′ 10.2″ E: 29° 28′ 6.6″	699 m	Emerging from extensive swamp
11	30 Nov.	Maridi dam	Kazana PS	N: 4° 53′ 14.7″ E: 29° 27′ 58″	696 m	Stagnant water
12	30 Nov.	Maridi	Foot crossing to Kazana 2	N: 4° 53′ 4.2″ E: 29° 28′ 4.8″	697 m	Flows through papyrus swamp
13	2 Dec.	Maridi	Temeregia crossing	N: 4° 55′ 8.5″ E: 29° 27′ 36.9″	683 m	Fast flowing, heavy algae lining on rocks
14	2 Dec.	Maridi	Hi-Taraba village	N: 4° 55′ 20.8″ E: 29° 27′ 43.9″	689 m	Dominated with swamp

15	2 Dec.	Munguo	Confluence with Maridi	N: 4° 56′ 21.3″ E: 29° 27′ 3.7″	674 m	Swampy with no flow
16	2 Dec.	Maridi	Dam spillway	N: 4° 53′ 41″ E: 29° 27′ 27.5″	696 m	Heavy breeding of *S. damnosum* ++++
17	3 Dec.	Maridi	Cugamal village	N: 4° 55′ 19.4″ E: 29° 27′ 38.8″	681 m	Stagnant water with papyrus
18	3 Dec.	Maridi	Jebel village	N: 4° 65′ 39.7″ E: 29° 27′ 15.2″	684 m	Stagnant water, swampy
19	3 Dec.	Maridi	Munguo village	N: 4° 55′ 57.8″ E: 29° 27′ 9.7″	681 m	Vast papyrus swamp
20	3 Dec.	Maridi	Rice garden site	N: 4° 56′ 3.8″ E: 29° 27′ 13.7″	683 m	Stagnant water and swampy
21	10 Dec.	Mabulindi	Juba main road	N: 4° 56′ 3.8″ E: 29° 27′ 13.7″	710 m	Poor flow
22	10 Dec.	Mabulindi	Araka main bridge	N: 4° 56′ 0.0″ E: 29° 29′ 32.2″	710 m	Extensive papyrus swamp.
23	10 Dec.	Maridi	Maridi 2 village	N: 4° 55′ 28.7″ E: 29° 27′ 30.6″	690 m	Poor flow
24	10 Dec.	Mabulindi	Rumbek bridge	N: 4° 58′ 29.4″ E: 29° 26′ 60.5″	674 m	Sluggish flow
25	10 Dec.	Munguo 1	Yambio road	N: 4° 54′ 49.9″ E: 29° 26′ 21.1″	702 m	Swampy with elephant grass
26	10 Dec.	Munguo 2	Yambio road	N: 4° 54′ 57.8″ E: 29° 26′ 1.3″	704m	Extensive swamp
